# Leprosy Classification, Clinical Features, Epidemiology, and Host Immunological Responses: Failure of Eradication in 2023

**DOI:** 10.7759/cureus.44767

**Published:** 2023-09-06

**Authors:** Jihad Alrehaili

**Affiliations:** 1 Pathology, Imam Mohammad Ibn Saud University, Riyadh , SAU

**Keywords:** nodule plaque, leprosy classification, erythematous skin lesions, lepromatous leprosy, mycobacterium leprae, leprosy

## Abstract

Leprosy is of big concern in the medical fraternity. Leprosy is also known as Hansen’s disease. It is a curable communicable disease that remains prevalent in most countries all over the globe. It is a chronic granulomatous infection commonly caused by *Mycobacterium leprae* and *Mycobacterium lepromatosis*, which mainly show an effect on the skin and peripheral nerves. To control the disease and minimize the impact of the disease, much effort has been put into it for decades. Nearly 0.2 million fresh cases were documented in 2017 worldwide in spite of being declared “eradicated” by the WHO in the year 2000. However, impressive achievements have been made in several countries, including India; still, we are lagging behind the ultimate goal of the final disappearance of leprosy. Extensive migration is a crucial element that may transmit leprosy to unaffected areas. Additionally, there are several areas in the USA where person-to-person leprosy transmission has been reported without a prior history of exposure. Recently, WHO instigated a new Global Leprosy Strategy 2021-2030, termed "Towards Zero Leprosy." In this article, we review the clinical features, leprosy epidemiology, transmission, classification, host immunological response, and diagnostic challenges.

## Introduction and background

Leprosy has a spectrum of varied clinical presentations. Leprosy was classified by Ridley and Jopling based on histological and immunological features into five types: tuberculoid (TT), borderline tuberculoid (BT), mid borderline (BB), borderline lepromatous (BL), and lepromatous leprosy (LL) [1,2]. The frequent lack of sensation on the skin, visible hypopigmented skin, hyperpigmented anesthetic, and hypoesthetic skin lesion are the main clinical presentations of leprosy. At the TT pole, there are some defined hypopigmented and anesthetic saucer-shaped lesions. It is associated with loss of sweating and lack of adnexal bodies. Since the patient is not immunocompromised and cell-mediated immunity (CMI) is good, lesions are usually small and solitary; leprosy may be cured by multidrug therapy (MDT) [1].

The BB form of leprosy is considered an unstable form, showing dimorphic punched-out lesions. There are erythematous plaques, which might be annular or circular, with externally diffused borders, well-defined internal structures showing multiple lesions, maculopapular, and nodules. The lepromatous pole of the spectrum is presented by multiple nodules and papules, with diffuse infiltrated skins, which result in madarosis and leonine facies [2]. A number of lesions are seen with bilateral symmetry. In the more severe disability, neural network involvement is observed. Diffused and nodule forms of LL have been seen [3,4].

Nerve damage in leprosy can vary, where the intradermal nerves can be involved in skin patches or the peripheral nerve trunk can present a large lesion [5]. Leprosy involves the superficial nervous system like the great auricular, median, ulnar, sural, posterior tibial, and superficial peroneal [6]. Neuropathy and associated disabilities are the chief medical outcomes of leprosy which is a huge medical concern worldwide. Nerve damage in leprosy can vary; the intradermal nerve can be involved in the cutaneous patch or the peripheral nerve trunk with a prominent lesion. These are clinically palpable and tender in the case of neuritis. Loss of sensation, touch, pain, and temperature assess the sensory impairment over the skin lesions. Neuronal injury in leprosy may present as weakness or may result in silent neuropathy, contracture, or atrophy [7,8]. Stocking and glove pattern of sensory damage is seen toward the lepromatous pole [9,10].

The polar forms of leprosy are stable while the borderline forms have an increased propensity to develop reactions. Patients with leprosy can present two major forms of reactions. Delayed-type hypersensitivity reaction is represented by a reversal or type 1 reaction, which is predominantly seen in cases of borderline leprosy [11,12]. Erythema nodosumleprosum or type 2 reaction is a type III hypersensitivity reaction with a sudden beginning of a severe inflammatory reaction because of the deposition of immune complexes [13,14].

## Review

Factors affecting leprosy epidemiology

Tuberculosis is a more common infection leading to severe morbidity. The epidemiological outline of leprosy is characteristically described in line with certain features, including age, classification, sex, rate of disability grade, and leprosy in children [15]. Sometimes it is difficult to eliminate it. The best approach to avoid leprosy transmission relies on early diagnosis and treatment. Endemic countries have faced such problems of delay in diagnosis, which resulted in episodes of disabilities. Poor technical training of healthcare providers is accountable for such negligence [16]. The advancements of précised complementary techniques would help shatter the transmission chain.

Risk Factors

**Figure 1:**
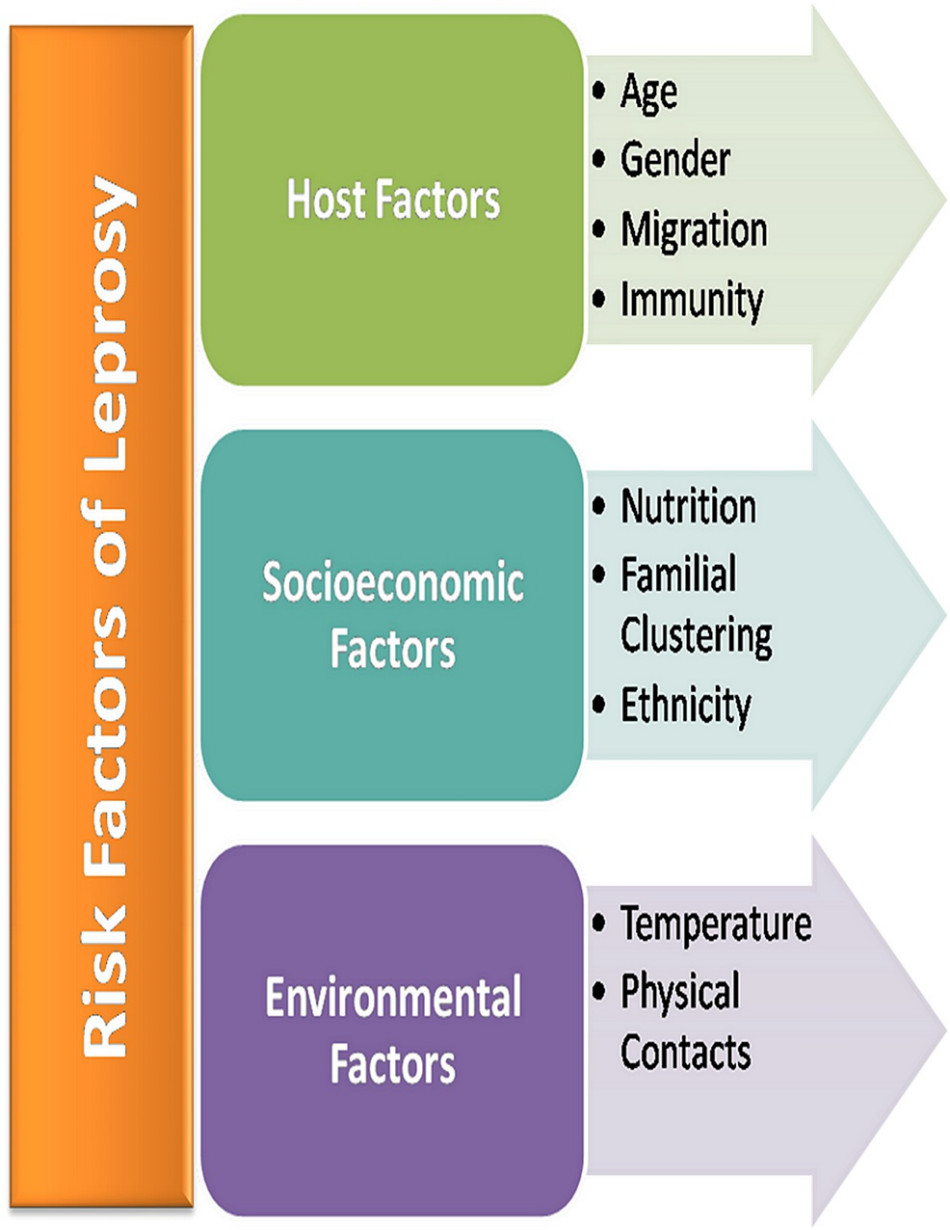
Various risk factors of leprosy

Inadequate housing conditions, proximity to the patient, overcrowding, improper diet (malnutrition), immunocompromised state (HIV), and rural inhabitation, all these factors lead to reduced cell-mediated immunity, and hence it is the ideal condition for infection whether by droplets or skin-to-skin contact (Figure 1) [15,16].

Host Factors

Age: Leprosy can be seen at any age; however, it more frequently occurs in the 20-30 years age group. The elevation of child leprosy cases has epidemiological importance in the population as it shows the presence of active spread of leprosy in the community. In lepromatous cases, the age distribution of cases commonly shows a later onset of disease as compared to non-lepromatous cases. 

Gender: Leprosy occurs in both genders. However, the female-to-male ratio is 1:2. Less number of female cases could be attributed to their meager mobility and decreased chances of contact [17].

Migration: Since there is migration of large numbers of the population from rural to urban areas, in recent years, cases of leprosy have been elevated in urban sectors. Urban slums have distinct geographic and population characteristics, common to all urban areas. The population density in the slums is very high (10,000-15,000/km^2^) where most of the people have migrated from far places like villages and tribal areas with poor living and hygiene conditions and highly compromised breathing space (8-10 or even more people sharing the same room) [18,19].

Contacts: Intensity and physical distance from an index LL case were directly associated with an increased risk of developing leprosy [20,21].

Ethnicity: Types of the disease vary between ethnic groups: In Micronesia, the frequency of disability is very low, whereas in China, it is very high. In the stumpy endemic region, the infection may be seen in the middle age. Involvement of the lower branches of the facial nerve occurs more often in China, but it is very rare elsewhere, and some reactional states such as the Lucio phenomenon occur mainly in the Americas. There are no groups immune to leprosy, although the rate of the disease may vary [22].

Genetic factors and susceptibility: Leprosy is a disease of very low infectivity and high morbidity. Only a few people exposed to infection develop clinical signs of the disease. Since several molecules are involved, they probably play an integral role in determining the immune response of the host to the infectious agent. Studies suggest that, among monozygotic (identical) twins, if one has leprosy, the other almost always has leprosy, while this was not the case with dizygotic twins. Monozygotic twins share all genes while dizygous twins share half the genes [23,24].

Immunity: The immunological status of an individual decides the probability of the occurrence of the leprosy disease. At the TT pole, there is strong cell-mediated immunity, very few bacilli, and localized lesions [25]. At the other pole, that is, the lepromatous one, there is a lack of cell-mediated immunity, a strong humoral response, disseminated disease, and a large no of bacilli [26]. Thus, TT patients can be thought to have shown maximum resistance whereas lepromatous patients have the least.

Familial clustering: The occurrence of leprosy can be seen because of genetic (close familial) relatedness or favorable environmental situations or by close contact with an affected family member. The chances of getting a familial cause of leprosy are higher in families where any lepromatous patient is there than those having no leprosy patient [27,28].

Nutrition: Malnourishment is a well-known cause of compromised immunity in people and results in a more vulnerable population to serious infectious diseases [29]. Poverty and lack of resources play a significant role in the rise of malnutrition in underdeveloped or developing countries leading to more cases of leprosy [30].

*Environmental Factors* 

Humidity favors the endurance of *Mycobacterium* spp. in different environmental settings. Bacterial cells remain alive for almost 46 days in wet soil samples and for nearly nine days in dry nasal secretions at room temperature. The risk of transmission increases with humid conditions [31].

Socioeconomic Factors

Leprosy is known to have been associated with multiple factors like lack of education, poverty, overcrowding, and lack of ventilation and personal hygiene, which favor transmission of the disease. The anxiety of leprosy, stigma, discrimination, and guilt coupled with the disease in the social network and baseless injustice concerning leprosy compel a patient to conceal the disease and result in delayed treatment, leading to the development of deformities and promoting the transmission of the disease. Even nowadays, despite the availability of enormous scientific advancements on leprosy, the myth is severely embedded in the minds of people at all levels of society that it is highly contagious and incurable [32].

Modes of transmission

Leprosy transmission pathways are not yet fully comprehended. Evidence stated that persons staying in close proximity with leprosy patients are at elevated risk, most expectedly via infectious air-dwelling droplets, formed by sneezing and coughing.

Infection via Inhalation

Moreover, it is known as a droplet infection. Currently, it is postulated to be the most common route of the spread of leprosy. During sneezing from a positive patient, millions of bacterial cells get released from nasal secretions. The major route of exit of the bacterium from an infected person is the respiratory tract primarily by the nose. Both skin and nasal secretions from cases of leprosy, which did not get any treatment, can transmit *M. leprae* to the environment. Such droplets/aerosols are deposited on either or both the skin and nasal epithelia of contact individuals with an ability of initiation of infection. Large numbers of bacilli are shed from the nose, especially when there are nasal ulcers [33].

Close Contact

The disease may also spread from skin-to-skin contact by shedding millions of bacilli from the torn skin and ulcers in a lepromatous patient [21].

In Utero Transmission

There are a handful of cases reported of leprosy in infants of very young age and most of them were having BT disease or indeterminate leprosy [34]. Only 50% of the mothers of these infants were found to have leprosy, which implied that half of the mothers had subclinical disease. High levels of IgG and IgM antibodies to *M. leprae *were found in infants born to lepromatous mothers [35]. Regardless of all this evidence in support of uterine transfer of infection, one seldom comes across affected babies of mothers with untreated lepromatous disease [36].

Ingestion of Breast Milk

It is observed that the breast milk of lepromatous mothers contains bacilli which are transmitted via the epithelial lining of the lactating mammary glands [37,38]. However, there is not any clear evidence that breast milk with viable leprosy bacilli acts as a source of infection.

Inoculation Following Trauma

There are a small number of cases in which leprosy lesions have been recorded after a thorn prick tattooing, vaccination injury or during dressing in a leprosy hospital, or any injury via a surgeon handling the leprosy patients [39].

Incubation Period

The latent period of the disease is variable and is unusually long. It can last from a few weeks to up to 20 years. However, the average incubation period of the disease is 2-7 years. Moreover, it has been observed that patients with paucibacillary (PB) leprosy have a shorter incubation period [40,41].

Case definition

As per the eighth meeting of the expert committee of WHO on leprosy held in 2010, a leprosy case is defined as someone who has one or more of the three chief signs [42,43]:

• erythematous skin lesions or hypopigmented skin with definite malfunctioning or lack of sensation; 

• peripheral nerve involvement, as depicted by explicit thickening on impaired sensory nerves; 

• positive skin smear for acid-fast bacilli (AFB).

Classification

**Figure 2:**
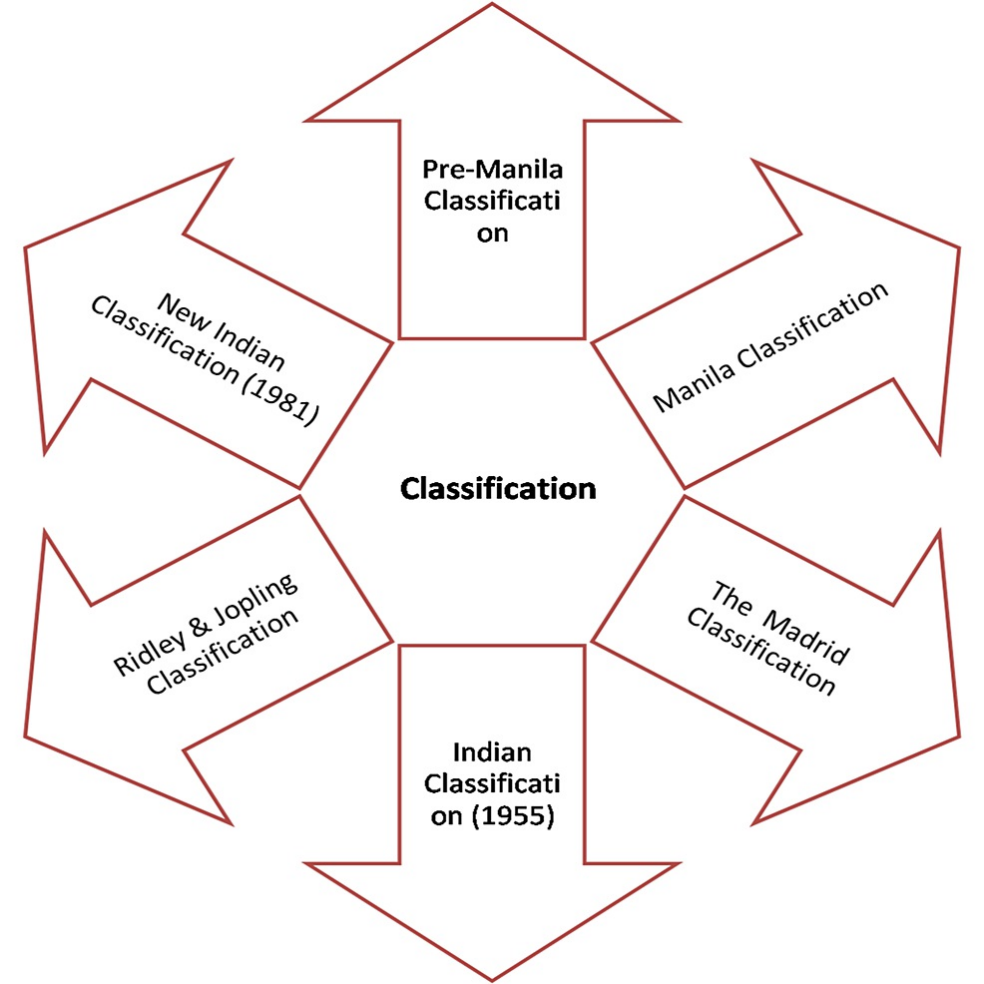
Classification of leprosy

The classification of leprosy is presented in Figure 2.

Pre-Manila Classifications

• Danielssen & Boeck (1847) [44]

• Danielssen & Boeck (1848) [45]

• Hansen &Looft (1895) [46]

• Neisser (1903) [45]

*The Manila Classification (1931)* 

The Leonard Wood Memorial held a round table conference (1931) in Manila [47], Philippines, and classified leprosy into the following:

• cutaneous (corresponding to the nodular of Hansen and Looft),

• neural (corresponding to the maculoanesthetic of Hansen and Looft),

• mixed.

The Madrid Classification (1953) [45]

• Lepromatous type

• Tuberculoid type

• Indeterminate group

• Borderline group

The Indian Classification (1955) [45]

• Tuberculoid (T)

• Lepromatous (L) 

• Maculoanesthetic (MA)

• Borderline (B) 

• Polyneuritic (P) 

• Indeterminate (I)

Ridley & Jopling Classification [48]

In 1966, they defined five groups based on clinical, bacteriological, immunological, and histological aspects.

• Tuberculoid leprosy

• Borderline tuberculoid

• Borderline

• Borderline lepromatous

• Lepromatous leprosy

New Indian Classification (1981) [49]

• Indeterminate (I)

• Tuberculoid (T)

• Borderline (B) 

• Lepromatous (L) 

• Polyneuritis (P)

In this classification, the maculoanesthetic form was clubbed to TT. The consequential five-group classification is known as the "New Indian classification of leprosy."

Clinical features of different types of leprosy

Leprosy mainly involves the skin and nerves while systemic involvement is present toward the lepromatous pole. The clinical features of leprosy are generally due to the response of the host to the bacilli rather than the direct damage from bacillary invasion [50,51].

Indeterminate Leprosy

The first skin lesion to appear is usually a small- to medium-sized hypopigmented patch situated mostly on the thigh and face, having vague edges with some loss of sensation [52,53]. As the disease progresses, similar patches appear all over the body. Hair growth and nerve function are not affected. They appear dry and often present with a wrinkled appearance. The diagnosis is confirmed by biopsy in which there is peri-neurovascular infiltration. AFBs are absent. Seventy-five percent of the indeterminate lesions are healed by themselves, while the rest become determinate, which enter in the range as definite types [54]. The prognosis of this type of leprosy is excellent. Reactions do not occur.

Tuberculoid Leprosy

It is a stable form and presents as a hypopigmented lesion with an erythematous border, with a single, stable, hairless plaque. Lesions are up to three in number, measuring <10 cm. A plaque with raised precise edges sloping inwards is known as the classic lesion. The surface is dry, hairless, anesthetic, and scaly, with a loss of sweating [15]. Typically, a lone peripheral nerve trunk is thickened near the lesion. AFBs are absent on slit skin smears. The lepromin test is positive suggesting good immunity. The prognosis is good.

BT Leprosy

Lesions resemble TT; however, they are quite large in number, bigger, and not well-defined [55]. The outer margin of the lesion slopes toward the normal skin. Characteristic finger-like projections known as pseudopodia or satellite lesions may be seen. The lesions are often scaly, dry plaques with reduced sensation. The nerves are asymmetrically thickened [56].

Borderline Leprosy

It is a highly immunologically unstable form of disease in the leprosy spectrum [12]. Patients can rapidly upgrade or downgrade toward either the TT form of the disease or the lepromatous pole. Dimorphic lesions are seen, which are characteristic of both TT and lepromatous types. Multiple, asymmetric lesions either as infiltrated papules, plaques, or sometimes even nodules are seen. The characteristic lesion is an annular plaque with a sloping outer margin and a well-defined inner margin presenting a characteristic Swiss cheese manifestation. The nerve involvement is inconsistent. The latter may be symmetrical or asymmetrical, if BB is reduced from BT or if BB is upgrading from BL, respectively. AFB is positive [56].

BL Leprosy

Lesions are multiple and symmetrical, often starting as hypopigmented macules with indistinct borders merging into the normal skin [57]. As the disease progresses, the macules become infiltrated and form plaques and nodules. The peripheral nerve trunks are thickened but the nerve injury is not as worrying as observed with BT. Patients with BL leprosy may show type 1 reactions or type 2 reactions. AFBs are strongly positive [56].

Lepromatous Leprosy

The cell-mediated immunity is harshly weakened leading to uncontrolled multiplication of bacilli. AFBs are highly positive. The lesions of LL are numerous with bilateral symmetry over the face, extremities, and trunk [58]. Lesions may present in any of the stages.

Early Macular Stage

Lesions during the early stage are mostly multiple macules that are slightly erythematous or hypopigmented, with indistinct borders, merging into the surrounding skin [59]. Sensations are normal.

Infiltrated Stage

Macules, if left untreated, may progress to develop induration which is more marked on the ear lobules and face [60].

Late Nodule Plaque stage

**Figure 3:**
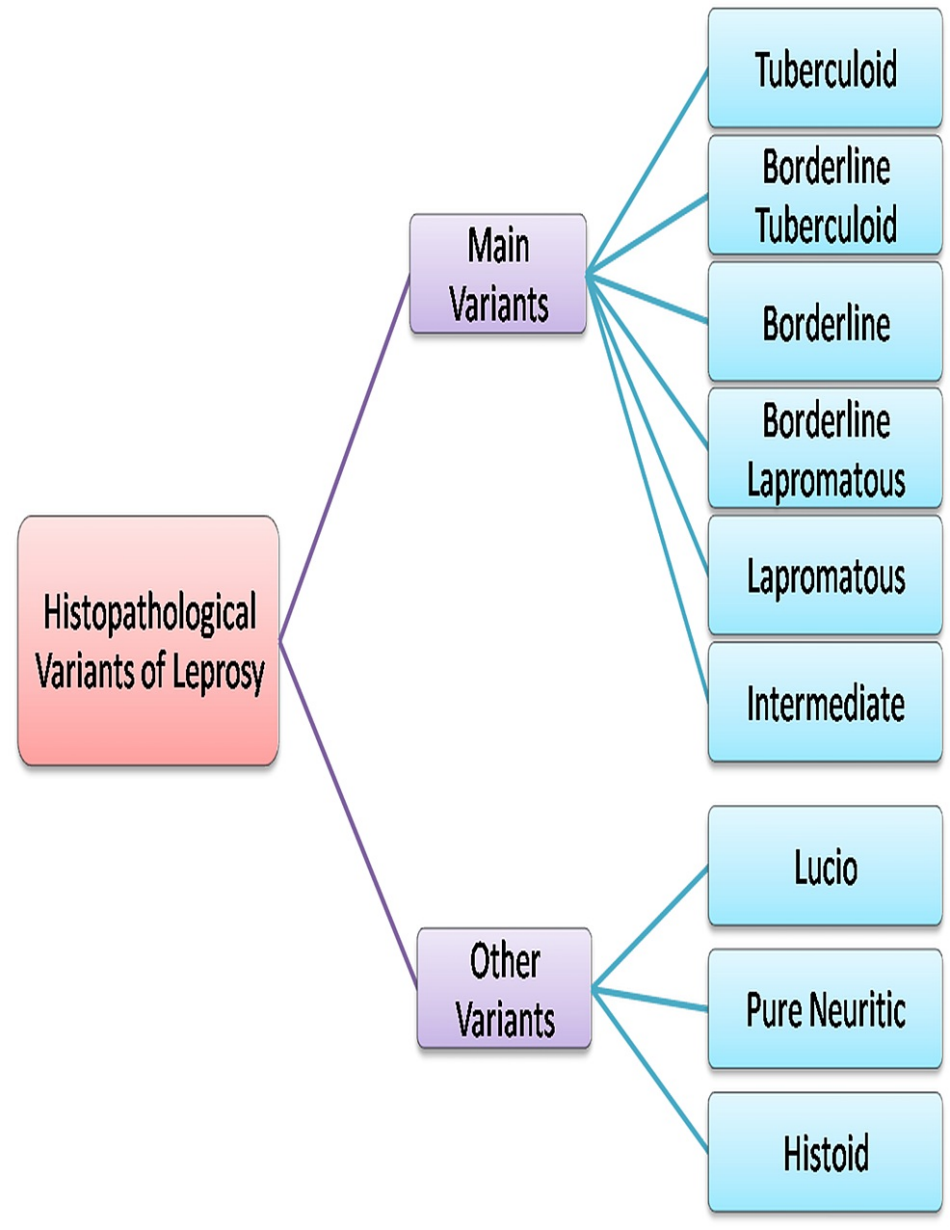
Lesion-presenting stages of leprosy.

Treatment is essential because if not treated properly, the induration will increase and stages of papules, even plaques or nodules, will result from the macular stage of progression [61]. The nodules first appear on the ear lobes and the buttocks, elbows, genitals, and fingers. The nodules may produce ulcers with a high bacterial load, making the disease extremely contagious. Involvement of the nose causes nasal stuffiness, crusting, and epistaxis. There may not be nerve thickening in the early stage of LL, but late cases show glove and stocking anesthesia. The prognosis of untreated LL is poor (Figure 3) [56].

The histopathological features of different types of leprosy [62,63]

*Tuberculoid Leprosy* 

It shows a granulomatous inflammatory infiltrate, with classic periadnexal and perineural allocation in the deep and superficial dermis. Granuloma is noncaseating, produced by epithelioid cells, lymphocytes, and Langhans cells. The epidermal features are frequently eroded and atrophic due to the granulomatous tissues. The perineurium of nerves is covered by lymphocytes. Some cases represent infiltrated nerves in granulomatous tissues. The bacillus is not detectable by bacteriological analysis, but the diagnosis is usually confirmed by a positive lepromin test, and granulomas are typically found on biopsy.

BT Leprosy

It shows an analogous infiltrate like TT. Epithelioid cells are vaguely matured, which represents the primary difference along with lymphocytes that are also present inside the granulomas. The epidermal region is not surely toughened by granuloma. Nerves are fairly puffed up with intraneural and perineural granulomas. Lymphocytes can be seen in the perineurium region. 

Borderline Leprosy

It also shows granulomas containing immature epithelioid cells. These granulomas are ill-defined. Lymphocytes are diffusely present in the absence of giant cells. Macrophages are present. Giant cells are usually not seen in this type of leprosy. The epidermis is atrophic. Nerves are not swollen.

BL Leprosy

Lymphocytes and macrophages constitute the infiltrate. It can be patchy, diffused, nodular, periadnexal, or perivascular but is segregated at all times from the epidermis to a narrow zone called the Grenz zone. Macrophages bear a frothy cytoplasm. Neurons contain a perineurium with "onion-skin" features surrounded by lymphocytes. Plasma cells are also there.

Lepromatous Leprosy

It shows foamy macrophages that are diffusely distributed in the dermis. Plasma cells and lymphocytes are seen scattered, and epithelioid cells are absent. Grenz zone and atrophic epidermis are typically present. There is a perineural collection of macrophages on onion-skin perineurium. AFBs are arranged in parallel or in the form of clusters, or as large masses called globi.

Other Variants

Lucio leprosy [64]: A diffused shape of polar LL, known as Lucio leprosy, is common in Mexico. *M. leprae* and the recently described *M. lepromatosis* are the causative bugs of Lucio leprosy. Skin is diffusely infiltrated, especially of hands and face, which gives a moon face impression; therefore, it is also known as "Leprabonita" or beautiful leprosy [56].

Lucio phenomenon: Lucio phenomenon is analogous to erythema nodosum leprosum (ENL) and is described by clear purpuric lesions which later progress to form ulcerations that heal with atrophic white scars [65,66].

Pure neuritic leprosy: Peripheral nerve trunk without any skin lesions is a characteristic feature of pure neuritic leprosy [8,67]. The clinical features include pain, nerve thickening, and tenderness. Clinically, it is likely to be TT or BT depending on the number of nerves involved [67].

Histoid leprosy: It is an atypical variant of LL with distinctive histopathological and clinical features [68]. It commonly occurs due to dapsone monotherapy or irregular treatment, or sometimes de novo. Clinically, it shows subcutaneous and cutaneous plaques and nodules with normal skin surroundings. Histopathologically, it shows multiple histiocytes with spindle shapes positioned in whorls, bands, or tight circles [69,70].

Diagnosis

Leprosy diagnosis is decided by the clinical symptoms and signs [71].

The skin lesions are generally hypopigmented, which may be numerous, or solitary, or sometimes erythematous nodules, macules, or papules. Failure of sensation is a normal characteristic. Another important feature of leprosy is the thickened neurons. Acid-fast, rod-shaped bacilli are also found in some cases, which are helpful in the diagnosis of leprosy disease.

Following are the features in which at least one should be shown by a patient suffering from leprosy in endemic countries [72,73]:

(i) The prepared smear should show the presence of *Mycobacterium* by Fite Faracco; 

(ii) permanent failure of sensation in an erythematous or hypopigmented skin patch; 

(iii) peripheral nerve thickness with impaired sensation.

Immunological response in leprosy [74]

Leprosy is considered the first disease to be classified based on the host's immune response. The clinical features of leprosy vary from one patient to another based on the spectrum of disease but more importantly on the type of host’s immune response to the bacteria. Both innate and acquired immune responses have been associated with leprosy, but the disease is typically described by the side of a Th1/Th2 response, where the Th1 response corresponds to the most restricted presentations and the Th2 to the most circulated ones. Leprosy immune response is associated with an increase in the inflammatory activity both in restricted and disseminated presentations, resulting in the worsening of previous symptoms or the advancement of new symptoms. This reaction was seen only in patients of the TT spectrum and not in those with LL, suggesting that the inflammatory response of the patient relied on the immune response of the host to the bacteria [74,75]. Patients with TT are decided by a relevant T-cell immune response, including interleukin-2 (IL-2), IL-4, IL-6, IL-10, interferon-γ (IFN-γ), tumor necrosis factor (TNF), and IL-17 and lymphotoxin, marked by some neural or cutaneous lesions with a few or no bacilli. In contrast, patients with LL show a superior humoral immune response, characterized by many lesions, elevated bacterial load, and reduced lymphocyte production.

Innate Immunity in Leprosy

**Figure 4:**
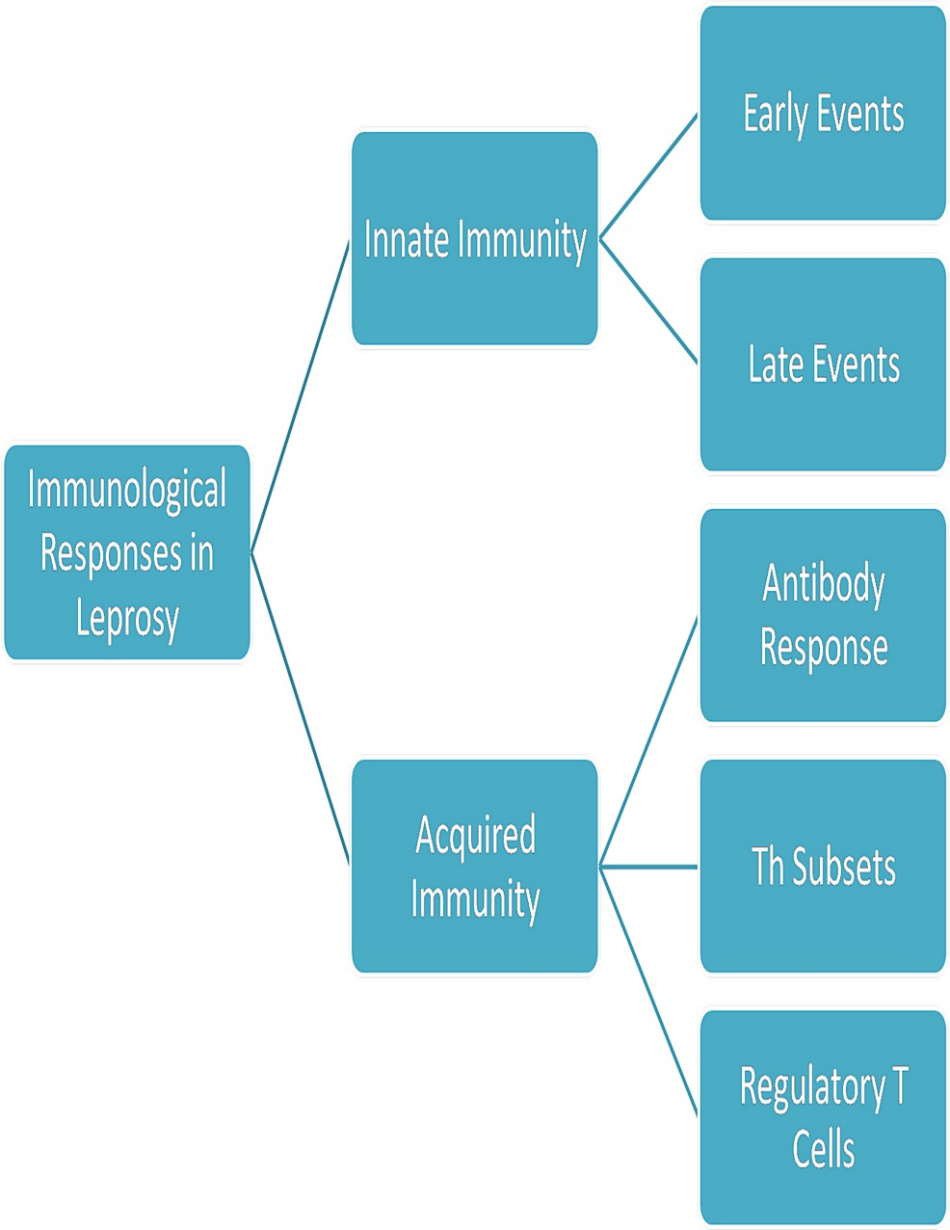
Immunological responses triggered by the host during leprosy

Dendritic cells and resident macrophages play a role in the early interaction between the host and pathogen at the site of infection [75]. Macrophages induce inflammatory intermediaries, which can further turn into a defined subpopulation of lymphocytes. This procedure builds a profile of adaptive and innate immune responses, which can control the infectious agents’ development. Consequently, the primary interaction between the macrophage and bacteria is critical for the final result of the infection (Figure 4) [76].

Early events of innate immunity: For the innate immune system, macrophages provide an important network of cells. They are the major producers of cytokines and are useful for not only innate immunity but also for adaptive immunity. Macrophages associated with tissues are one of the prime host cells targeted by Mycobacteria [77]. The initial step of phagocytosis of *M. leprae* is mediated with the help of three complement receptors. These are CR1 (Complement Receptor 1), CR3, and CR4. *M. leprae*-macrophage interactions are instigated at the same time by PRRs (pattern recognition receptors), which identify the pathogens associated with general molecular structures [78]. Such interaction engages PRRs like NOD2 (nucleotide-binding oligomerization domain-containing protein 2) and TLRs (toll-like receptors) [79]. Activation of the latter resulted in the elevated release of both TNF and IL-12. It has also been reported that the expressions of TLR1 and TLR2 are upregulated in TT patients’ skin biopsies compared to those of lepromatous ones [79]. NOD-like receptors are another significant cluster of PRRs, which have a considerable associated role in *M. leprae* infection pertaining to innate immune responses [80].

Autophagy is a severe cellular mechanism for monitoring cytosol refinement and can also compel pathogens for degradation by phagolysosomes. It is well known that autophagy acts as a key role player to control the spread and replication of mycobacterium [81]. *M. leprae* induces the synthesis of lipid bodies (LBs) in the host cell, which has been related to leprosy pathogenesis and contributed to continuous infection [82]. Hormone-sensitive lipase is decreased by *M. leprae*, which facilitates the protection of the lipid-rich surroundings, which are suitable for the intracellular survival of the organism [83]. An autophagic complex is formed that transports LBs to lysosomes, where hydrolysis of LB occurs to produce ABCA1-dependent free cholesterol efflux inside foamy macrophages [84]. The secretion and expression of insulin-like growth factor-I are increased while *M. leprae* infection causes proliferative and apoptotic activities in macrophages and Schwann cells (SC) [85].

Late events of innate immunity: For growth and virulence, mycobacteria need iron, just like other bacteria, as it is a crucial nutrient for microorganism survival. The communication of SC with *M. leprae* is governed by adhesins localized in the bacterial cell wall, which interact with the extracellular matrix of the host cells. The *M. leprae* PGL-I, HLP, and other cell wall components are competent to interact with the domain G of the α-2 laminin chain of the host extracellular matrix proteins. The function of the α-2 laminin chain is considered as a bacterial cell receptor, which interacts with the α-dystroglycan localized in the basal lamina, which encircles the SC, resulting in engulfed pathogens [86]. Interactions of *M. leprae* and SC were carried out by several PRRs as seen in the macrophage cases. TLR6 is crucial in SC to induce the production of LB and PGE2 promoting intracellular Mycobacterium survival. SC are demyelinated during *M. leprae* infection [87].

Intracellular iron concentration is sensed during iron metabolism regulation, which modulates the storage and uptake of the latter accordingly. For protection against oxidative stress, iron storage proteins are very essential [88].

The initial defense against *M. leprae* is carried out by the innate immune response, which is afterward followed by an acquired immune response. Leprosy bacilli first enter and then live in macrophages, SCs, and dendritic cells. Phagocytosis is mediated by certain receptors to complements, which are CR1, CR3, and CR4. CR3 is used to identify a specific cell wall lipid of *M. leprae*, i.e., PGL-1 [89]. These TLRs and complement receptors are present on dendritic cells and macrophages and are important for the identification of pathogenic microorganisms. TLR-2 and TLR-4 identify the *Mycobacterium* spp. and stimulate monocytes and liberate IL-12 [79]. In TT skin lesions, TLR-1 and TLR-2 are more effectively expressed.

Acquired Immune Response

Interactions of macrophages, dendritic cells, lymphocytes, cytokines from T cells, and antibodies from B cells are included in the acquired immune response. TT patients show unnoticeable antibodies but a good T-cell response. LL patients on the other hand show a large number of antibodies but no T-cell-mediated immune response.

Antibody responses: There is a polyclonal B-cell response seen in the lepromatous spectrum. Humoral response to PGL-1 and its conjugates is shown in 90-95% of lepromatous patients and TT patients accounted for 25-60% [90].

Th subsets: Early studies showed that TT patients had a Th1 subset, while Th2 was predominant in LL patients and was also constant with the T-cell and B-cell responses seen in lepromatous and TT, respectively, but other studies illustrated that a few leprosy patients also showed Th profile with both IFN-γ (TH1) and IL-4 (TH2) [91]. Instead of monocytes, dendritic cells induce cytokine discharge in the same patients [92]. It indicated that the movement of Th1 and Th2 in TT and LL, respectively, was not absolute. Hence, multiple unidentified factors are responsible for the antigen unresponsiveness [93].

Regulatory T cells: Two regulatory T cells have recently been studied extensively. IL-17-producing Th17 cells have been recognized in humans, and are considered as the chief cytokines. In type 2 reactions, Th17 cells are well involved [94]. It has been reported that in patients who are unable to build up a T helper response, Th17 may be a salvage pathway or when the polarization of T helper cells has not been set up [95].

Leprosy Reactions

T-cell responses to *Mycobacterium* are activated in type 1 reactions, leading to inflammation of the nerves and skin. There is increased lymphoproliferation and an increased release of proinflammatory cytokines [96,97]. Immune complex deposition leads to ENL in the vessels [98]. Release of IFN gamma and IL-12, triggered by antigen-specific T-cell activation, has been reported [99]. An elevated level of IL-4, IL-6, and IL-8 has also been reported in leprosy [100]. Hence, there is a temporary augmentation of T-cell responses in LL patients, which keeps on going even after a drop in clinical features [101].

Hence, TT patients are found to show a Th1 proﬁle with interleukin-2 and interferon-γ, while LL patients have a Th2 proﬁle with IL-10 and IL-4. Type 1 reaction also shows a Th1 profile, while there is a shift from Th2 to Th1 when LL patients develop a type 2/ENL reaction. However, many studies show that this dual observation could not provide an overall justification, as a lot of TT and lepromatous patients demonstrated the occurrence of non-polarized Th0 subsets of CD-4 cells liberating both IL-4 and IFN-γ. LL patients during Type 2/ENL reactions illustrate a shift to a Th1 proﬁle with the production of IFN-γ and reduced levels of IL-4, suggesting dysregulation of cytokines that may cause tissue damage. 

Th17 population is another CD4+ Th subset, which is associated with inﬂammation that confirms the variance in leprosy. Th17 subset is lower in leprosy patients compared to the highest in healthy individuals exposed to diseases, suggesting its significance in innate immunity. Non-polarized Th0 subset harboring leprosy patients showed the presence of Th17 cells. Consequently, it is clear that the Th17 subset plays a crucial function in the immune response and hence provides an alternating pathway for eliminating bacilli in the onset as well as later stages of the disease. 

A rise in Th17 cells was seen in patients of both types of reactional leprosy in comparison to the non-reactional ones with identical symptoms, as demonstrated by the presence of IL-17F and IL-17A in CD4 cells. IL-17 and the signature cytokine Th17 subset contribute to inﬂammation in leprosy lesions along with IFN-γ of the Th1 subset. IL-17 may be used as a surrogate marker because it is detected in the serum of healthy individual contacts along with patients of TT. IL-17 can also be utilized for monitoring vaccine efficacy and treatment response. IL-17 contributes to nerve damage as growth factors influence the peripheral nerves, which is a characteristic feature of this disease. This requires further investigations into whether the latter is an alternate defense mechanism or a rescue pathway. 

In the Th22 response, IL-22 is reported as one of the key implicated proteins, which acts as an anti-inflammatory or proinflammatory cytokine, relied upon in the response to the disease [102]. The presence of IL-22 in LL may trigger a mechanism during the tissue response, which may result in tissue hyperplasia. IL-22 cytokine controls the effect of growth factors like FGF-b, which is significant for the production of extracellular matrix, propagation of keratinocytes, and stimulation of angiogenesis [103]. IL-17 and IL-22 levels are increased in TT while they are low or absent in patients along the lepromatous spectrum. Both types of patients with reactional leprosy demonstrated a rise in IL-17A, IL-17F, and IL-22 levels.

Epidemiology of leprosy

Leprosy is endemic in tropical countries, especially in underdeveloped or developing countries. Since the introduction of MDT in the early 1980s, the prevalence of this disease has decreased significantly, while there are still 105 countries in the world that have an endemic strain, specifically located in Southeast Asia, the Americas, Africa, the Eastern Pacific, and the Western Mediterranean, which have a high prevalence of cases. In 2011, there have been 219,075 new cases detected in the world. There were 181,941 new cases recorded and there was a prevalence of 0.34 cases per 10,000 inhabitants in the first quarter of 2012. Ninety-five percent of cases were reported from 16 countries (mainly in Asia, Africa, and South America) (Tables 1, 2) [104].

**Table 1:** Regions contributing data to WHO in 2021

Data retrieved from the various regions by WHO in 2021		
	Regions	Number of new cases in 2021
AFR (African Region)	37	21201
AMR (American Region)	27	19826
EMR (Eastern Mediterranean Region)	21	3588
EUR (European Region)	16	14
WPR (Western Pacific Region)	31	2480
SEAR (South East Asia Region)	11	93485
Total	143	140594

**Table 2:** Annual leprosy statistics from WHO in 2021

2021 annual leprosy statistics from WHO	
Countries providing the data	143
Global priority countries	23
Global leprosy burden of 23 countries	140,594
Prevalence	17.83/million population
Patients treated	133,802
New cases	140,594
Grade 2 disabilities	8492
Child cases	9052
Female cases	55,349 (39.3%)

Leprosy epidemics have affected and terrified the population of all continents, and the ancient civilizations of China, Egypt, and India regarded leprosy as a disease with an irrepressible nature, causing disfigurement, and being highly contagious. The epidemiological data from some countries, including India, should be interpreted cautiously, since disease elimination goals were achieved by a number of criteria, including altering the definition of case, excluding recurrent cases from prevalence rates, excluding cases of treatment dropout from active records, treating PB patients with a single dose, shortened treatment durations, etc. As a result, the number of new cases reported fell significantly [105]. In Brazil, leprosy prevalence has declined markedly since 2000. The detection rate has dropped gradually in recent years, probably as a result of the increase in the accessibility of primary care services for patients [106]. Since this is the primary endemic monitoring indicator in Brazil, reducing leprosy cases among children under 15 years of age is a priority. These cases indicate recent transmission with an active infection focus and a high endemic area, revealing operational deficiencies. Typically, the source of the infection is close to the patient, which is why an analysis of the contacts the patient has had with them will likely provide the best indication. The peak number of cases detected among people under 15 years of age occurred in 2003 when 4,181 cases were detected, resulting in a detection coefficient of 7.98 per 100,000 inhabitants. Since then, the rate of detection has been decreasing; in 2011, there were 2,420 new cases detected, resulting in a detection coefficient of 5.22 per 100,000 inhabitants [107]. In Saudi Arabia, a total of 242 leprosy cases have been reported over the past 10 years, with 67% of cases occurring in individuals between the ages of 15 and 44. Males accounted for more than 77% of all cases, while non-Saudi nationals, dominated by Indians, accounted for 57.4% of the total count [108].

The lack of knowledge and access to specific treatment in some regions contribute to the late diagnosis of leprosy, which may, in turn, lead to physical disability, an indicator used to measure the quality of services. There was a reduction in the number of physical disabilities in leprosy cases in 2011 due to the larger number of early diagnoses in the country, but 2,165 of these cases had grade 2 disabilities. This could be attributed to a hidden prevalence of leprosy, that is, an undetected reservoir of infections maintained by epidemiological and operational factors (Table 3) [104,109].

**Table 3:** Number of new cases reported in the global priority countries from 2017 to 2021.

Year	Cases
2017	201,289
2018	199,400
2019	193,904
2020	122,227
2021	133,008

The main reason for this is the lack of awareness and knowledge among people about leprosy and its symptoms, which are often neglected or left untreated until it is too late. Additionally, poverty and lack of access to medical care further contribute to the problem.

Clinical studies on leprosy 

The histologic characteristic feature of leprosy is the granuloma, possessing macrophages, which have been invaded by the pathogen *M. leprae* and lymphocytes. Various species of mycobacteria are responsible for leprosy, where some are pathogenic like *M. leprae*, *M. tuberculosis*, *M. scrofulaceum*, and *M. marinum* and some are non-pathogenic, e.g. *M. fortuitum* and *M. indicus pranii*. The treatment of multibacillary cases of leprosy with MDT consisted of 12 doses of a combination of dapsone, rifampicin, and clofazimine. Some of the mycobacterial species grow fast in vitro and some are slow growers [5]. 

In 2012, Da Motta-Passos et al. conducted a study on 48 untreated leprosy patients (13 females and 35 males) [110]. They looked for reduced RNA expression of IL-17A in leprosy patients by using ELISA and PCR. They observed that levels of IL-17A mRNA were significantly decreased in cases in comparison to controls. In 2012, Martiniuk F et al. conducted a study to analyze the Th17 levels in ENL subjects who were on thalidomide [111]. Levels of IL-17A were persistently upregulated and treatment with thalidomide had no effect on IL-17A levels. A reduction in amounts of IL-17B as well as IL-17E along with an increase in IL-17C after thalidomide treatment was noted. Hence, it was concluded that TH17 cells play a crucial role in the immunopathogenesis of ENL.

In 2013, Chaman Saini et al. conducted a study on 37 newly diagnosed leprosy patients [95]. This was done to assess the role of T helper 17 cells in leprosy. ELISA was used for cytokine secretion from PBMC and PCR for evaluating gene expression. IL-17 isoforms showed significantly higher levels in healthy contacts and TT (mean 101.9 pg/ml ± 26.28) as compared to LL (mean 45.5 pg/ml ± 22.27). IL-22 levels did not show a significant increase in BT (mean 561.4 pg/ml ± 118) as compared to LL (mean 633.1 pg/ml ± 89.18). They concluded that in the more unresponsive forms of TT, CD4 IL-17 cells play a part in adaptive immunity.

In 2013, Marwa Abdallah et al. recruited 43 untreated patients with different types of leprosy and 43 controls to look for the levels of IL-17 and IL-4 [112]. This was done using ELISA. F.B. de Almeida-Neto et al. published a study to examine the association between TH-17 cells, interleukin-17, and interferon-gamma in 23 leprosy patients and household contacts of leprosy. Peripheral blood samples were analyzed to identify TH-17 cells, interleukin-17, and IFN gamma using immunocytochemistry, and the relationships between all the groups were established. The study confirmed the active participation of TH-17 cells and IL-17 in the immunology of leprosy. A positive association was also recognized between IFN gamma and IL-17, as well as the segregation of the frequency of these cells between individuals who have PB forms and those individuals with MB forms of the disease.

In 2014, Attia E AS et al. published a study in which they evaluated serum IL-17 and IL-22 levels in 43 untreated leprosy cases and 40 healthy volunteers [113]. Patients were investigated in accordance with histopathological examination, clinical examination, and SSS. ELISA was done on serum samples. IL-10 and TGF-beta were significantly higher in patients as compared to controls, while IL-17 was significantly lower (median in cases: 19 pg/ml and median in control: 37.5 pg/ml) while the difference in IL-22 levels was insignificant (median in cases: 75 pg/ml and median in control: 72.5 pg/ml). They found very low levels of serum IL-17 in cases, but the levels of IL-22 were high only in ENL patients when compared to controls. They concluded that leprosy has inadequate secretion of IL-17 but IL-22 was not affected.

In 2016, Saini C et al. conducted a study to analyze the role of CD4+ subsets of Th17 cells and CD25+FOXP3+ regulatory T cells (Tregs) in leprosy type 1 and type 2 reactions [95]. Cases were 30 patients of leprosy with reactions and 36 new stable leprotic patients formed the control group. ELISA was done for the assessment of IL-17A and 1L-17F, IL-22, IL-23A, IL6, IFN-gamma, and TGF-beta. Both type 1 and type 2 reactions are associated with a significant increase of IL17-A levels (mean in RR 219.8 ± 28.07 pg/ml, mean in ENL 650 ± 195.2 pg/ml) compared to matching stable forms of leprosy (mean in BT 101.9 ± 2.6 pg/ml, mean in LL 45.5 ± 22.7 pg/ml). Mean IL-22 levels in RR were 492.8 ± 115 pg/ml, 96.72 ± 93 pg/ml in ENL, 561 ± 118 pg/ml in BT, and 633.1 ± 89.1 pg/ml in LL. 

In 2017, Edessa Negera et al. conducted a study for the estimation of Treg cells in patients with Type II reaction for which 46 cases with ENL reaction and 31 LL patients without reaction as controls were recruited [114]. Blood samples were taken at three times, once before, during, and then after treatment of ENL with prednisolone. PBMCs were separated and then used for immunophenotyping of regulatory T-cells. They concluded that in ENL, there is an increase in the CD4/CD8 T-cell ratio, a decrease in the percentage of regulatory T cells, and an increase in IL-17-producing T-cells. 

In 2018, Costa MB et al. conducted a study on 74 leprosy patients to evaluate Th17 cytokines in paired samples of leprosy in both types of leprosy reactions [115]. T regulatory cells were also evaluated. They concluded that before and during reactions in paired skin biopsies, there was an increase in the number of Treg cells during T1R. This suggested its important role in the control of intense inflammation and cellular immunity. It was noted that Treg levels were normal and there was an elevation in the amount of IL-17 in type II reactions.

In 2019, Siti Sakdiah et al. published a study to compare IL-17 levels in leprosy and non-leprosy patients on 40 leprosy patients and 40 non-leprosy controls using IL-17 ELISA kits [116]. The median value of IL-17 for non-leprosy patients was 47.86 pg/ml. The median value of IL-17 in lepers was 102.86 pg/ml. There were significant differences in IL-17 levels in lepers and non-lepers. IL-17 was higher in leprosy patients than in non-leprosy patients.

## Conclusions

Due to uncultivated causative agents of the disease, molecular epidemiology investigations are notoriously challenging in leprosy. Despite noteworthy progress in understanding the biology of leprosy bacilli through molecular approaches, the accurate mechanism of disease transmission is still unclear. We proposed more combined investigations on immunological and genetic aspects to clarify the development, onset, and underlying mechanisms of leprosy. These outcomes may open a door to understanding a part of the immunological course of this disease, its clinical features, and its epidemiology for better control where more research is needed to be done.
